# Pregnancy in MNGIE: a clinical and metabolic honeymoon

**DOI:** 10.1002/acn3.51202

**Published:** 2020-11-07

**Authors:** Pauline Pappalardo, Jean‐François Benoist, Bridget E. Bax, Clarisse Carra‐Dallière, Cecilia Marelli, Michele Levene, Laetitia Begue, Anne Rolland, Nicolas Flori, François Rivier, Catherine Blanchet, Arnold Munnich, Romain Altwegg, Pierre Meyer, Agathe Roubertie

**Affiliations:** ^1^ Département de Neuropédiatrie CHU Gui de Chauliac Montpellier France; ^2^ Laboratoire de Biochimie métabolomique Hôpital Necker APHP Paris France; ^3^ Molecular and Clinical Sciences St George’s University of London London SW17 0RE UK; ^4^ Département de Neurologie CHU Gui de Chauliac Inserm U1198 MMDN Univ Montpellier Montpellier France; ^5^ Département de Gynéco‐obstétrique CHU Arnaud de Villeneuve Montpellier France; ^6^ Département de Pédiatrie CHU Nantes Nantes France; ^7^ Département des Soins de Support Institut du Cancer de Montpellier (ICM) Montpellier France; ^8^ U1046 INSERM UMR9214 CNRS Université de Montpellier Montpellier France; ^9^ Service d'ORL et Chirurgie Cervico Faciale CHU Gui de Chauliac Montpellier France; ^10^ INSERM UMR1163 Institut Imagine Paris France; ^11^ Département de Gastroentérologie CHU St Eloi Montpellier France; ^12^ Institut des Neurosciences de Montpellier INSERM U1051 Université de Montpellier Montpellier France

## Abstract

Mitochondrial neurogastrointestinal encephalomyopathy (MNGIE) is an inherited disease caused by a deficiency in thymidine phosphorylase and characterized by elevated systemic deoxyribonucleotides and gastrointestinal (GI) and neurological manifestations. We report the clinical and biochemical manifestations that were evaluated in a single patient before, during, and after pregnancy, over a period of 7 years. GI symptoms significantly improved, and plasma deoxyribonucleotide concentrations decreased during pregnancy. Within days after delivery, the patient’s digestive symptoms recurred, coinciding with a rapid increase in plasma deoxyribonucleotide concentrations. We hypothesize that the clinico‐metabolic improvements could be attributed to the enzyme replacement action of the placental thymidine phosphorylase.

## INTRODUCTION

Increasing numbers of patients with inborn errors of metabolism (IEM) reach adult reproductive age. IEM present a challenge for the health of child‐bearing women and their fetus and specifically, patients with mitochondrial diseases have been reported to be at increased risk of complications during pregnancy and labor.^1^, ^2^ Mitochondrial neurogastrointestinal encephalomyopathy (MNGIE, ORPHA#298; OMIM#603041) is a rare mitochondrial disease caused by autosomal recessive mutations in the nuclear *TYMP* gene which encodes for thymidine phosphorylase (TP), a cytosolic enzyme involved in the degradation of the deoxyribonucleosides thymidine (dThy) and deoxyuridine (dUrd). TP deficiency is responsible of a progressive and fatal degenerative disease with an onset between the first and second decades of life. It is characterized by gastrointestinal (GI) and neurological manifestations, including cachexia, GI dysmotility, peripheral neuropathy, leukoencephalopathy, ophthalmoplegia, and ptosis. Plasma and urine dThy and dUrd levels are increased compared to undetectable levels found in unaffected individuals.^3^, ^4^, ^5^, ^6^ Here we report clinical and metabolic improvements during pregnancy in a patient with MNGIE.

## Methods

The study was conducted in accordance with the Declaration of Helsinki and was approved by the local ethical committee review board. Patient informed consent was obtained. Clinical and metabolic manifestations were evaluated in a single patient before, during and after pregnancy, for a period of 7 years.

## RESULTS

**Figure 1:**
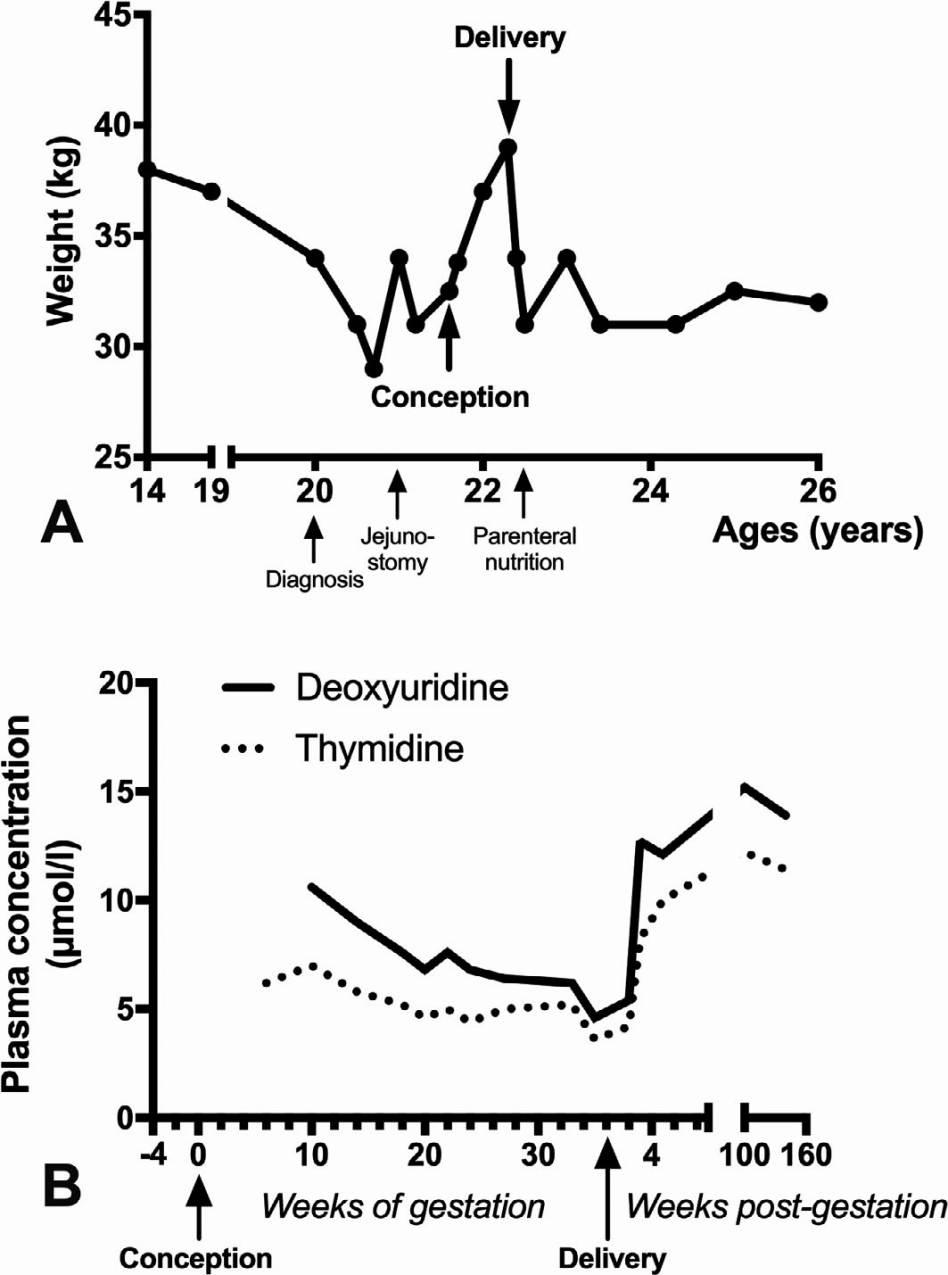
A: Body weight of the patient between 14 years of age until last follow‐up at 26; B: plasma concentrations of deoxyribonucleosides during pregnancy.

The patient was the first child of non‐consanguineous parents. She accomplished postgraduate level studies and was considered healthy, but petite. At 19 years, an episode of vomiting, diarrhea, and abdominal pain with a 5 kg weight loss, but without biological signs of dehydration persisting for 10 days were reported (Table 1). Thereafter, she complained of daily episodes of vomiting, bloating, abdominal pain, and constipation. Despite fractionation of meals and various medications, early satiety and weight loss persisted (Fig. 1A). A diagnosis of MNGIE was confirmed at 20 years of age with null lymphocyte TP activity and compound heterozygote biallelic pathogenic variants in the *TYMP* gene identified. The c.215‐1G> C variant was inherited from her healthy father and the c.1211T> C variant was inherited form her healthy mother.^7^

A jejunostomy provided a 4‐kg weight gain, but no improvement in her severe digestive symptoms were noted. Fatigability increased during exacerbation of her GI symptoms. Chronic neuropathic pain and GI symptoms were partially improved by clonazepam, paracetamol, amitriptyline, tiapride, and lactulose associated with L‐carnitine supplementation.

At 21.7 years, the patient was referred for delayed menstruation related to an unexpected pregnancy. From the third week of gestation (WOG), GI symptoms improved. All medications except L‐carnitine were discontinued. From the 6th WOG onwards, vomiting and abdominal pain disappeared, and oral feeding increased for the first time in 2 years. From 9 WOG, constipation dramatically improved and painful limb dysesthesia disappeared. At 24 WOG, the patient was treated with nefedipine for premature labor. Weight gain (Fig. 1A), pregnancy follow‐up, and fetus growth parameters were normal, with the patient reporting overall well‐being during pregnancy. Plasma dThy and dUrd levels progressively decreased during pregnancy (Fig. 1B).

Instrumental vaginal delivery of a male child occurred at 36 WOG who displayed mild intrauterine growth retardation (weight 2410 g, height 44 cm, cranial perimeter 32 cm). The child is presently 4.5 years old, and has achieved normal developmental milestones.

Two days after delivery, the patient’s GI symptoms recurred with major feeding difficulties even with jejunostomy. The patient rapidly lost body weight and plasma dThy and dUrd levels increased (Fig. 1A and B), B). Treatment with clonazepam, gabapentin, decorenone, and occasional nefopam was initiated. A central catheter was implanted 3 months after delivery to complement parenteral nutrition but required removal due to sepsis or thrombosis. Jejunostomy became progressively ineffective and neurosensorial impairment, particularly fatigue worsened (Table 1). Now at 26 years of age, the patient is independent in daily living activities and caring for her child, despite fatigue.

Table 1 shows semi‐quantitative scores modeling GI and neurological functional symptoms that highlighted their improvement during pregnancy. Quality of life improvement was clearly reported by the patient, and retrospective assessment in daily living activities was achieved using the Karnofsky performance score.

## Discussion

Here we report on a young woman whose cardinal symptoms of MNGIE dramatically improved throughout pregnancy. Plasma deoxyribonucleoside levels gradually decreased during pregnancy, but returned to levels characteristic of MNGIE after delivery. Two days later, symptoms recurred, with disease progressing in concordance with MNGIE.

To our knowledge, a spontaneous improvement, that may be considered as a clinical and metabolic honeymoon in our patient, has not been previously reported in patients with MNGIE, whose disease is characterized by a progressive deterioration. Indeed, their metabolite derangement persists unchanged until the end‐stage of the disease without spontaneous decrease or significant fluctuations.^8^ Persistent reduction of dThd and dUrd plasmatic levels has only been reported following therapeutic interventions aimed at replacing the deficient enzyme,TP.^4^ The clinical and metabolic changes observed in our patient should be ascribed therefore to her pregnancy; we hypothesize that accumulating metabolites are translocated from the maternal circulation to the placenta via nucleoside transporters, where they are cleared by placental TP throughout pregnancy. The role of the fetus in this metabolism, however, remains unknown.

The retrospective collection of clinical and metabolic data is a limitation of our study. To address this issue, we built a semiquantitative score for modeling functional symptoms that highlighted their improvement during pregnancy. Quality of life and retrospective assessment in daily living activities based on Karnofsky performance score, clearly improved in our patient.

Mitochondrial deoxynucleoside triphosphates (dNTPs) are synthetized by the cytosolic de novo pathway in proliferating cells or by the salvage pathway in postmitotic tissues and quiescent cells. Mitochondrial dNTP pool equilibrium is critical for correct mtDNA replication.^9^ TP is a mitochondrial enzyme that plays a pivotal role in the pyrimidine nucleoside salvage metabolic pathway as it catalyzes the reversible phosphorylation of dThy and dUrd to 2‐deoxyribose 1‐phosphate and their respective bases, thymine and uracil. A deficiency in TP results in a systemic accumulation of dThy and dUrd and their incorporation in the mitochondrial pyrimidine salvage pathway leading to dNTP pool imbalance that ultimately leads to mtDNA deletions, somatic point mutation and depletion.^10^

Phenotypic manifestations of the disease develop when a threshold level of mtDNA damage is reached, and results in energy production failure in postmitotic tissues.^3^

Nucleosides translocate between extracellular and cellular compartments, via specific transporters belonging to the human nucleoside transporter (hNT) family. NTs are highly expressed in placenta where they enable prominent passive transport of nucleosides from the maternal circulation to the trophoblast.^11^ TP is also strongly expressed in placenta.^12^

The toxicity of accumulated dThy and dUrd is considered a prominent pathophysiological factor in MNGIE, and therapeutic strategies aimed at reducing their systemic overload have been developed, including hemodialysis, platelet transfusion, hematopoietic stem cell or liver transplantation, and enzyme replacement therapy.^3^, ^4^, ^5^, ^13^ However, the management of MNGIE patients remains extremely challenging owing to the high rates of complications associated with these approaches.^4^, ^5^, ^14^ In conclusion, the observation of a marked clinical and metabolic improvement of the disease during pregnancy represents a physiological proof of concept of the systemic and widespread benefit of TP activity restoration/replacement performed by a unique targeted tissue, and support the perspective of investigational therapies like enzyme replacement therapy using autologous erythrocyte encapsulated thymidine phosphorylase or gene therapy.^4^, ^8^

## Conflict of Interest

All the authors confirm that there is no conflict of interest to declare.

**Table 1:** Clinical and paraclinical features of the patient and their course.

Signs or symptoms (age at onset in years; FS : functional score; WOF: week of gestation)			
Features	Before pregnancy	During pregnancy	Follow‐up after pregnancy
Gastrointestinal			
	poor weight gain (adolescence ? weight 37kg, Height 157 cm, body mass Index 15 at 19 years) (FS:1)	7 kg weight gain throughout pregnancy (FS:0)	persistent at last follow‐up (FS:1)
	weight loss (19 y) (FS:1)	No (FS: 0)	persistent at last follow‐up (FS:1)
	vomiting (19 y) (FS:1)	resolution from 6WOG (FS:0)	persistent at last follow‐up (FS:1)
	gastroparesis with early satiety (19 y) (FS:1)	improved from 3 WOG (FS:1)	persistent at last follow‐up (FS:1)
	bloating (19 y) (FS:1)	resolution from 6WOG (FS:0)	persistent at last follow‐up(FS:1)
	abdominal pain (19 y) (FS:1)	resolution from 6WOG (FS:0)	persistent at last follow‐up(FS:1)
	severe constipation spontaneous stools/10 days (19 y) (FS:1)	improvement from 9WOG (spontaneous stool emission every other day) (FS:1)	persistent at last follow‐up(FS:1)
	jejunostomy feeding (21 y) (FS:1)	oral feeding (FS:0)	jejunostomy removal (22.5 y) total parenteral feeding (23 y) (FS:2)
Neurological			
	fatigue (19 y) (FS:1)	improved from 6WOG (FS:1)	increased at last follow‐up (FS:1)
	4 limbs abolished reflexes( 19 y)	Stable	stable at last follow‐up
	distal limb thermo‐algic sensation dysfunction ( 19y)	Stable	increased at last follow‐up
	distal painful paresthesia (20 y) (FS:1)	resolution from 9WOG (FS:0)	Increased at last follow‐up (FS:1)
	nerve conduction velocities : sensorimotor demyelinating neuropathy (20 y)	not assessed	stable at last follow‐up
Neuro‐sensorial			
	right‐sided neurosensorineural hearing loss (absent acoustic evoked otoemissions, 70 dB right hearing thresholds at auditory evoked brainstem potentials) (20 y) left hearing impairment (21.5 y) (FS:1)	not assessed, stable disability (FS:1)	right cophosis and left moderate sensorineural down sloping hearing loss (26.5 y) (FS:1)
	normal ophthalmological assessment (FS:0)	not assessed, no disability (FS:0)	ophthalmoparesis without optic neuropathy (25.5 y) (FS:1)
Brain Magnetic Resonance Imaging			
	leucoencephalopathy with T2 hypersignal of the cerebral white matter and striatum and midbrain (20 y)	not assessed	stable (24 y)
Other			
	No cardiac or myopathic involvement, normal plasma lactate levels and lipid profile up to last follow‐up (26 y)		
Total functional score	11	4	13
Karnofsky performance score	60%	90%	40%

A simple scoring system of GI and neurological functional symptoms was achieved by attributing for each a score 0 when the symptom was absent and 1 when the symptom was present ; for feeding, we used 0 for oral feeding,1 for enteral tube feeding, and 2 for parenteral nutrition. Total score highlights the course of the symptoms before, during, and after pregnancy. Clinical examination features and paraclinical data (electrophysiology, brain MRI, biology) were not included in this score. Autonomy in daily living activities was assessed using Karnofsky performance score.
